# Assessing the Validity of the Fellow Eye as an Internal Control in Early-Phase Clinical Trials for Myopic Chorioretinal Atrophy

**DOI:** 10.3390/jcm15082997

**Published:** 2026-04-15

**Authors:** Norimichi Nagano, Eisaku Kanemori, Yoshio Hirano, Takahiro Hojo, Yukinori Sakaeda, Takaaki Yuguchi, Soichiro Kuwayama, Shuntaro Ogura, Masayo Kimura, Hiroshi Morita, Kohei Uemura, Tsutomu Yasukawa

**Affiliations:** 1 Graduate School of Pharmaceutical Sciences, The University of Tokyo, Tokyo 113-0033, Japan; naganonorimichi@g.ecc.u-tokyo.ac.jp; 2 Department of Ophthalmology and Visual Science, Nagoya City University Graduate School of Medical Sciences, Nagoya 467-8601, Japan; 3 Interfaculty Initiative in Information Studies, Graduate School of Interdisciplinary Information Studies, The University of Tokyo, Tokyo 113-0033, Japan

**Keywords:** comparative study, geographic atrophy, myopia, myopic chorioretinal atrophy, retinal pigment epithelium

## Abstract

**Background/Objectives:** Age-related macular degeneration, particularly geographic atrophy, is a major cause of irreversible vision loss and shares pathological features with myopic chorioretinal atrophy (CRA). This study was designed as an exploratory methodological analysis to evaluate the feasibility of using the fellow eye as an internal control in early-phase clinical trials for myopic CRA. **Methods:** This exploratory and methodological retrospective study included eight patients (16 eyes) with myopic CRA who visited the Department of Ophthalmology at Nagoya City University Hospital between January 2010 and August 2023. Atrophic areas in both eyes were measured, and the longitudinal changes were analyzed. Three mixed-effects models were compared to assess the impact of inter-individual and inter-ocular variability on atrophic area progression. Subsequently, fixed-effects and mixed-effects models were compared using the Akaike Information Criterion (AIC). Finally, the square root of the variance ratio was calculated to quantify the contribution of inter-ocular variability to atrophic area progression. **Results:** In all eyes, the square root of the atrophic area increased over time. The model including random intercepts and slopes for each eye nested within patients had the lowest AIC of 69.4, suggesting that accounting for both inter-individual and inter-ocular variability improved model accuracy. The mixed-effects model had a lower AIC than the fixed-effects model, indicating a better fit. The square root of the variance ratio was 0.34 in the mixed-effects model, indicating that the inter-ocular variability was lower than the inter-individual variability, though it remained appreciable. **Conclusions:** This study quantitatively supports the feasibility and methodological validity of inter-ocular comparison designs for early-phase clinical trials in myopic CRA.

## 1. Introduction

Progressive macular atrophy is a major cause of irreversible central vision loss in multiple retinal disorders, including non-neovascular age-related macular degeneration (AMD) and pathologic myopia [1]. In geographic atrophy (GA) secondary to AMD, progressive loss of the retinal pigment epithelium (RPE) and photoreceptors, often accompanied by choriocapillaris impairment, underlies enlarging atrophic lesions and functional decline, making accurate quantification of atrophy progression a central issue in interventional trials [2]. Methodological frameworks for monitoring atrophic progression using ocular imaging and for optimizing clinical trial design are increasingly relevant across atrophic macular diseases. Myopic chorioretinal atrophy (CRA) is a serious late-stage complication of pathological myopia [3,4,5]. Myopic CRA is often bilateral and progressive, eventually resulting in legal blindness and severe impairment of central vision. Although the pathogenesis of myopic CRA is not fully elucidated, axial length elongation related to myopia can disrupt and deform the scleral architecture. It can also cause retinal and choroidal thinning and decrease the density of RPE cells with a breach of Bruch’s membrane, an underlying stiff structure [6,7,8]. The progression of the atrophic area over time can lead to irreversible vision loss. Myopic choroidal neovascularization (CNV) can be treated with several intravitreal injections of anti-vascular endothelial growth factor (VEGF) agents [9,10,11]. However, myopic CRA may develop within a decade after the onset of myopic CNV [12,13]. Once myopic CRA has progressed to this stage, interventions are ineffective, leading to severe vision loss [9]. In pathologic myopia, myopic CRA can affect the macula, resulting in central scotoma and progressive vision loss, which adversely affects the patient’s quality of life [14,15].

In the absence of established treatments for myopic CRA, regenerative medicine is expected to be one of the most promising future therapeutic options. Owing to recent advances in regenerative medicine, several therapeutic approaches have been proposed to slow or halt the progression of myopic CRA. Our group is currently conducting a phase 1/2a first-in-human clinical trial of novel human allogeneic adipose tissue-derived mesenchymal stem cell sheets (PAL-222) transplantation for myopic CRA (PAMyCA study) [16]. In parallel, therapeutic development for GA secondary to AMD has also accelerated in recent years, further highlighting the need for robust and sensitive approaches to quantify atrophic progression as structural endpoints [2,17]. To evaluate the efficacy of such interventions in the clinical trial, appropriate study designs that account for inter-individual variability and ensure statistical validity are essential. However, assessing CRA progression remains challenging because of substantial inter-individual variability, which necessitates large sample sizes in conventional parallel-arm trial designs. An alternative approach is inter-ocular comparison, in which one eye receives treatment and the fellow eye serves as a control. In early-phase clinical trials for bilateral ocular diseases, treatment is generally administered to the eye with poorer visual function due to ethical and clinical considerations. This approach minimizes potential risks while enabling therapeutic assessment. Therefore, inter-ocular comparisons using the untreated fellow eye as a control can represent a practical and ethical study design for early-phase clinical trials with limited sample sizes.

The progression of myopic CRA is influenced by various systemic and ocular factors, including age, blood pressure, axial length, intraocular pressure, choroidal thickness, and lifestyle variables such as outdoor activity, diet, and smoking [18,19,20,21,22]. Because it is difficult to control these factors across individuals, inter-ocular comparisons provide a more reliable and biologically meaningful approach than comparisons involving unrelated subjects. However, challenges remain in defining an appropriate control group in clinical trials evaluating efficacy. Although this inter-ocular design effectively controls for systemic, genetic, and lifestyle confounding factors, it depends on the assumption that inter-ocular variability is less than inter-individual variability. Notably, in GA secondary to AMD, intra-individual inter-ocular symmetry has been reported despite wide inter-individual variability, supporting the conceptual rationale for within-patient comparisons in progressive atrophic disease [23]. Despite its practical and ethical appeal, the validity of using the fellow eye as a control in evaluating myopic CRA progression has not been quantitatively examined. Specifically, it remains unclear whether the inter-ocular difference in the progression is sufficiently small to justify this approach.

This study aimed to evaluate the validity of an inter-ocular comparison design to assess myopic CRA progression in early-phase clinical trials where sample sizes are limited. We analyzed longitudinal data on the atrophic area of both eyes of eight patients and estimated progression slopes using fixed-effects and mixed-effects models to quantify inter-ocular variability, thereby determining whether the fellow eye could reasonably serve as an internal control. In the absence of established evaluation methods, this study quantitatively provides a clinically applicable approach to assess therapeutic efficacy, which may contribute to the future development of treatment options for myopic CRA.

## 2. Materials and Methods

### 2.1. Study Design

Given the relatively low prevalence of eligible patients and the slow progression rate of myopic CRA, a retrospective longitudinal design using existing clinical imaging data was adopted to efficiently evaluate inter-ocular variability in the progression of the atrophic area. This study was designed as an exploratory and methodological analysis to assess inter-ocular variability and the validity of using the fellow eye as an internal control in early-phase clinical trials for myopic CRA.

### 2.2. Overview of Patient and Measurement Characteristics

Participants aged 20 years or older were selected based on the presence of myopic CRA and the availability of longitudinal imaging data sufficient to assess disease progression, rather than a specific range of spherical equivalent or gender. A total of eight patients (16 eyes) were included in the analysis. Of these, one patient was male (12.5%) and seven were female (87.5%). The mean age was 71.6 ± 12.4 years, with a median of 74.5 years, ranging from 47 to 86. All patients had bilateral myopic CRA. Regarding ocular comorbidities, eyes with uncontrolled glaucoma were excluded. Two eyes had normal-tension glaucoma, one eye had secondary glaucoma, and the remaining 13 eyes had no ocular comorbidities. Thirteen eyes were pseudophakic, while three eyes were phakic; one phakic eye had a cataract. Atrophic area measurements were performed twice in four patients (eight eyes) and three times in four patients (eight eyes). The mean spherical equivalent was −8.75 D, ranging from −3.3 to −15.1 D. Patient and measurement characteristics are summarized in Table 1.

### 2.3. Atrophic Area Analysis

This retrospective observational study was conducted using clinical imaging data of patients with myopic CRA. A total of 48 fundus autofluorescence images were selected from the eyes of eight patients with myopic CRA who visited the Department of Ophthalmology at Nagoya City University Hospital on two or three occasions between January 2010 and August 2023. After anonymization of personal information in the images, two evaluators measured the area of the atrophic lesion (mm^2^) using image analysis software (RegionFinder, version 2.4; Heidelberg Engineering GmbH, Heidelberg, Germany). To evaluate the progression of atrophic areas, their mean values (calculated as the average of the measurements by Evaluator 1 and Evaluator 2) and the square roots of the mean atrophic areas (mm) were calculated for each eye at each time point. The square roots of the atrophic areas were plotted against time, and consecutive time points were connected with straight lines to visualize the change over time for the left and right eyes of each patient.

### 2.4. Evaluation of the Influence of Inter-Individual and Inter-Ocular Variability

To evaluate the influence of inter-individual and inter-ocular variability on the progression of the atrophic area, we constructed and compared three mixed-effects models. Model 1 included a random intercept for each patient; Model 2 included a random intercept and random slope for each patient; Model 3 included a random intercept and random slope for each eye nested within patient. For each model, we calculated the Akaike Information Criterion (AIC) and the fixed-effects slope estimate. AIC is a metric used to evaluate the goodness-of-fit of statistical models based on a given dataset with a penalty for model complexity [24,25,26]. A lower AIC value indicates a better-fitting model. In all models, time (in years) was used as the independent variable, calculated as (measurement date − first measurement date)/365.25, and the square root of the atrophic area as the dependent variable.

### 2.5. Evaluation of Inter-Ocular Variability in Atrophic Area Progression Using Variance Ratio

We then constructed two statistical models to evaluate inter-ocular variability in the progression of the square root of the atrophic area. The first was a fixed-effects model that included fixed effects for inter-individual and inter-ocular differences and their interaction with time. The second model was a mixed-effects model that incorporated random effects for inter-individual and inter-ocular variability in slopes over time. Both models were fitted to the data, and the slopes of the square root of the atrophic area were estimated. We then calculated the square root of the variance ratio, defined as the square root of the mean square for inter-ocular variability within patients divided by the mean square for inter-individual variability. We evaluated whether inter-ocular variability represented a substantial source of variability in atrophic area progression. To confirm the consistency of the results, the same statistical analyses were repeated as a sensitivity analysis using an alternative population excluding an atypical case.

### 2.6. Statistical Analysis

Given the exploratory nature of this study and the limited sample size, no a priori sample size calculation was performed. The primary variables analyzed were continuous measurements obtained from ocular imaging. Statistical analyses were conducted descriptively to evaluate inter-ocular differences within individual patients. The distribution of continuous variables was examined descriptively.

## 3. Results

### 3.1. Atrophic Area Analysis

**Figure 1:**
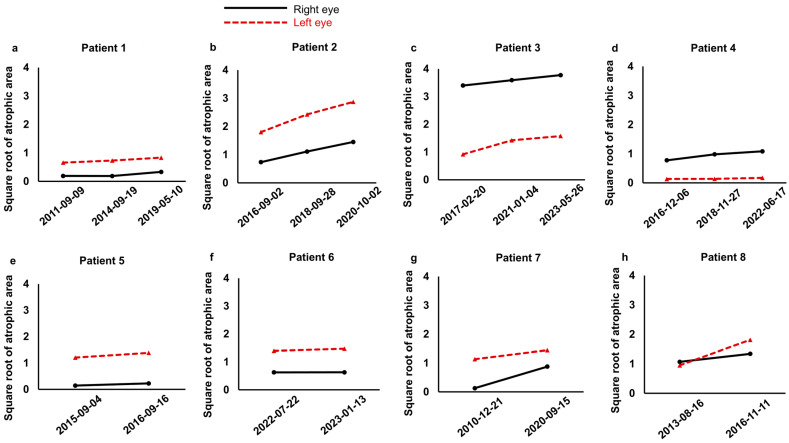
Time course of the square root of the atrophic area in both eyes of individual patients. (**a**–**h**) Longitudinal changes in the square root of the atrophic area for Patients 1–8, respectively. Each line represents one eye, with the square root of the measured atrophic area plotted at each time point and connected with straight lines to show progression. The right eye is shown as a black solid line with circles, and the left eye as a red dashed line with triangles.

Table 2 summarizes atrophic area measurements at each time point by two evaluators, including mean values and the square roots of the mean atrophic areas. Measurements from both evaluators were generally consistent across time points, showing progressive enlargement of the atrophic area. No substantial systematic discrepancies were observed between the two evaluators, supporting the reliability of the measurements. Figure 1 shows the change over time in the square root of the atrophic area in both eyes of each patient. In all eyes, the square root of the atrophic area increased over time. Most patients exhibited similar progression patterns between fellow eyes, supporting the assumption that inter-ocular variability is lower in magnitude compared to inter-individual variability.

### 3.2. Influence of Inter-Individual and Inter-Ocular Variability

Table 3 presents the AIC value and the fixed-effect slope estimate for each model. Among the three models tested, Model 3, which included random intercepts and slopes for each eye nested within patients, yielded the lowest AIC, indicating the best fit to the data. This result suggests that accounting for both inter-individual and inter-ocular variability provides a more accurate estimate of the progression of the atrophic area. Furthermore, the fixed-effect slope estimates were similar across all three models, indicating that the overall rate of progression was consistent regardless of the random-effects structures.

### 3.3. Slopes of the Square Root of the Atrophic Area and Variance Ratio Between Inter-Ocular and Inter-Individual Variabilities

**Figure 2:**
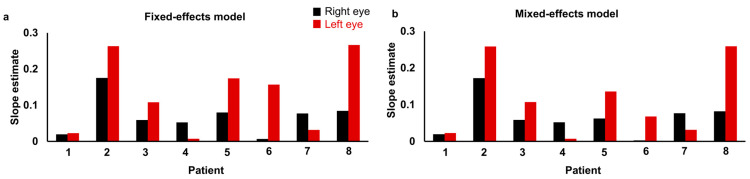
Comparison of individual slope estimates for the square root of the atrophic area from (**a**) fixed-effects and (**b**) mixed-effects models. Right eyes are shown in black bars and left eyes in red bars. Each bar represents the estimated slope for one eye of each patient under each model.

The fixed-effects model yielded an AIC of 19.2, whereas the mixed-effects model yielded a substantially lower AIC of 1.7, indicating that the mixed-effects model provided a better fit to the data (Table 4). The estimated slopes of the square root of the atrophic area, derived from both the fixed-effects and mixed-effects models, are presented in Table 4 and Figure 2. Except for Patient 6, the estimated slopes were comparable between the two models, indicating consistent progression estimates.

The square root of the variance ratio was calculated to quantify the relative contribution of inter-ocular variability to the overall variability in atrophic area progression. The values were 0.50 for the fixed-effects model and 0.34 for the mixed-effects model (Table 5), suggesting that inter-ocular variability is lower compared with inter-individual variability. These results indicate that the progression of atrophic areas between fellow eyes within the same patient is more similar than those across different patients.

Substantial differences in estimated slopes were observed between the two models for both eyes in Patient 6 (Table 4), likely due to the limited data points and the shorter observation period. To assess the consistency of the results, we repeated the analysis excluding Patient 6. In this analysis, the fixed-effects model yielded an AIC of 22.1, while the mixed-effects model yielded a lower AIC of 6.9, similarly indicating better model fit (Table 6). The square root of the variance ratio was 0.35 for the fixed-effects model and 0.34 for the mixed-effects model (Table 6), consistently confirming that inter-ocular variability is lower compared with inter-individual variability.

## 4. Discussion

To our knowledge, evidence specifically addressing inter-ocular variability in the progression of myopic CRA from a methodological perspective remains limited. However, the use of the fellow eye as an internal control has been widely adopted in ophthalmic clinical research, particularly in early-phase trials, to minimize inter-individual variability and to enhance statistical efficiency in small cohorts [27,28,29,30]. In this context, the present study provides quantitative evidence supporting the feasibility of a within-subject, inter-ocular comparison approach in myopic CRA.

In the present study, longitudinal changes in atrophic areas from eight patients were analyzed to investigate the contribution of inter-ocular and inter-individual variability (Table 1 and Table 2, and Figure 1). Based on these data, we compared three mixed-effects models to evaluate their impact on progression estimates (Table 3). Of the three models, model 3, which included random intercepts and slopes for each eye nested within patients, had the lowest AIC value of 69.4, indicating the best fit. This result suggests that accounting for both inter-individual and inter-ocular variability improves model accuracy. We next fitted the fixed-effects and mixed-effects models to the data and calculated their AIC values to evaluate model performance (Table 4). The mixed-effects model resulted in a substantially lower AIC of 1.7, indicating a better fit. We then estimated the slope of the square root of the atrophic area using the fixed-effects model and the mixed-effects model (Table 4 and Figure 2). The square root of the variance ratio serves as an interpretable index of inter-ocular variability relative to inter-individual variability. Higher values indicate that inter-ocular variability contributes substantially to overall variability and requires careful consideration in statistical analysis, whereas lower values suggest minimal inter-ocular differences, supporting the validity of using the fellow eye as an internal control. The square root of the variance ratio was relatively low at 0.34 in the mixed-effects models (Table 5 and Table 6), indicating that fellow eyes tend to show similar progression in myopic CRA. The square root of the variance ratio remained unchanged at 0.34 even with the inclusion of an atypical case of Patient 6, reinforcing that inter-ocular variability is lower in magnitude than inter-individual variability. These results support the feasibility of using the fellow eye as an internal control in early-phase clinical trials with limited sample sizes, thereby minimizing inter-individual confounding and supporting the appropriateness of the inter-ocular comparison design.

Our study examined inter-ocular and inter-individual variability in the progression of myopic CRA using a series of fixed-effects and mixed-effects models. By analyzing longitudinal data from both eyes of each patient, we quantified this variability using the square root of the variance ratio. Although this exact metric has not been widely reported, related methodologies—such as the square root transformation of lesion area to improve modeling of progression [31] and the evaluation of inter-ocular differences in neuro-ophthalmologic conditions [32]—support the relevance of inter-ocular comparisons in clinical research. Furthermore, the lower AIC values of the mixed-effects models and the consistent results after excluding Patient 6 support the validity of the inter-ocular comparison approach (Table 5 and Table 6).

In our analysis, the mixed-effects model consistently yielded lower AIC values than fixed-effects models, both with and without the inclusion of Patient 6, demonstrating superior model fit (Table 5 and Table 6). In mixed-effects models, individual slope estimates are often “shrunk” toward the overall mean, a phenomenon known as shrinkage estimation [33,34,35]. This effect is particularly pronounced when the number of observations per subject is limited or when individual estimates deviate substantially from the population mean. In our study, we observed this shrinkage effect (Table 4), indicating that the mixed-effects model effectively stabilized individual slope estimates by borrowing strength from the overall population. These findings support the application of mixed-effects models in studies with limited longitudinal data and reinforce the finding of low inter-ocular variability.

Therefore, within-subject crossover designs with randomized allocation of treatment to either eye effectively eliminate laterality bias and balance confounding factors [36,37,38]. In situations where randomization is not feasible due to ethical considerations, treating the eye with poorer vision and using the fellow eye as a control can still reduce bias in favor of the intervention. Such designs have been successfully implemented in studies of AMD [29]. Our findings suggest a generally high degree of inter-ocular similarity in the progression of myopic CRA, although a certain degree of inter-ocular variability cannot be disregarded. While an inter-ocular comparison design minimizes inter-individual variability and offers a practical approach for clinical trials, inter-ocular differences—whether due to asymmetrical axial length, localized pathology, or measurement error—may still influence outcomes. Therefore, these factors should be carefully considered in the design and analysis of clinical trials. The results in this study highlight that inter-individual variability plays a greater role than inter-ocular variability in determining the progression of myopic CRA. Consequently, using the fellow eye as an internal control can substantially reduce variability and improve statistical efficiency in clinical trials. This is particularly important in early-phase clinical trials with limited sample sizes evaluating novel regenerative treatments, where subject recruitment is challenging and variability in progression rates may obscure treatment effects. The inter-ocular comparison design, in which one eye receives treatment and the fellow eye serves as a control, offers distinct advantages. This design inherently controls for systemic confounding factors such as age, genetics, and overall health status, as both eyes share the same biological environment. By minimizing inter-individual variability, it thereby enables more precise evaluation of treatment effects on the progression of atrophic areas. We are currently conducting a phase 1/2a first-in-human clinical trial of novel human allogeneic adipose tissue-derived mesenchymal stem cell sheets (PAL-222) transplantation for myopic CRA [16]. In this early-phase clinical trial, the fellow eye is used as an internal control, thereby minimizing inter-individual confounding and supporting the use of the inter-ocular comparison design. This approach is critical for accurately assessing the therapeutic efficacy of new interventions in our clinical trial and also represents a broadly applicable and practical strategy for evaluating treatment effects in future clinical studies for myopic CRA.

Although this methodological study provides insights into inter-ocular variability, several limitations should be acknowledged. First, the sample size was small due in part to the relatively low prevalence of myopic CRA and the limited number of eligible patients, which precludes statistically powered hypothesis testing. The findings should be interpreted with caution, given the small sample size and exploratory design, and their generalizability remains limited. Larger, confirmatory studies with sufficient statistical power will be required to validate the present observations. The second limitation of this study is the inclusion of eyes with glaucoma. Although eyes with uncontrolled glaucoma were excluded, the potential influence of coexisting glaucoma cannot be completely ruled out. Future studies with larger sample sizes and more rigorous exclusion of glaucoma will be required. Third, the observational periods and the number of measurement points varied among patients, which may have introduced variability in slope estimates. However, mixed-effects modeling, which is well-suited for longitudinal data with uneven observation intervals, was used to appropriately account for such differences [35]. In addition, the degree of myopia was relatively wide, and the potential influence of spherical equivalent on inter-ocular variability could not be fully evaluated in this small cohort. The uneven gender distribution may limit the generalizability of the findings. Although inter-individual comparisons mitigate the influence of age-related factors, the wide age range may limit the generalizability of the findings. These factors may have introduced bias in the estimation of progression rates and should be considered when interpreting this methodological study. Lastly, although this study proposes a potential method for evaluating therapeutic efficacy in myopic CRA, further validation with larger and more diverse cohorts, ideally in prospective interventional studies, will be required to address these limitations, confirm the findings, and establish clinical utility. Despite these limitations, our study provides novel insights into the relative contribution of inter-ocular and inter-individual variability in myopic CRA progression. In the absence of established evaluation methods, this study offers a scientifically valid and clinically applicable approach for efficacy assessment, thereby contributing to the future development of treatment options for myopic CRA.

## 5. Conclusions

This exploratory and methodological study demonstrates the feasibility of using the fellow eye as an internal control in early-phase clinical trials for myopic CRA. The observed inter-ocular variance ratio of 0.34 indicates that within-subject inter-ocular variability is substantially smaller than inter-individual variability, supporting the methodological validity of an inter-ocular comparison design.

## Figures and Tables

**Table 1:** Overview of patient and measurement characteristics.

Number of Patients		8 Patients (16 Eyes)
Sex (%)		Male:1 (12.5%)/Female: 7 (87.5%)
Age (years)	mean ± SD	71.6 ± 12.4
	median (range)	74.5 (47 to 86)
Ocular comorbidities		Normal-tension glaucoma: 2 eyes Secondary glaucoma: 1 eye None: 13 eyes
Lens status		Pseudophakic eyes: 13 eyes Phakic eyes: 3 eyes (cataract: 1 eye)
Number of measurement points		Twice: 4 patients (8 eyes) Three times: 4 patients (8 eyes)
Spherical equivalent (D)	mean ± SD (range)	−8.75 ± 3.6 D (−3.3 to −15.1 D)

This table summarizes the clinical and measurement characteristics of the patients, including the number of patients, sex, age, ocular comorbidities, lens status, number of measurement points and spherical equivalent. SD: standard deviation.

**Table 2:** Atrophic area measurements by two evaluators at each time point, with mean values and the square root values.

Patient ID	Laterality	Time Point	Evaluator 1	Evaluator 2	Mean	Square Root of the Mean Atrophic Area
Patient 1	Right	2011-09-09	0.045	0.028	0.0365	0.1910
		2014-09-19	0.037	0.033	0.0350	0.1871
		2019-05-10	0.142	0.079	0.1105	0.3324
	Left	2011-09-09	0.413	0.455	0.4340	0.6588
		2014-09-19	0.479	0.596	0.5375	0.7331
		2019-05-10	0.697	0.695	0.6960	0.8343
Patient 2	Right	2016-09-02	0.585	0.496	0.5405	0.7352
		2018-09-28	1.315	1.151	1.2330	1.1104
		2020-10-02	2.190	2.022	2.1060	1.4512
	Left	2016-09-02	3.278	3.208	3.2430	1.8008
		2018-09-28	6.142	5.646	5.8940	2.4278
		2020-10-02	8.440	8.077	8.2585	2.8738
Patient 3	Right	2017-02-20	11.007	12.102	11.5545	3.3992
		2021-01-04	13.111	12.691	12.9010	3.5918
		2023-05-26	14.256	14.237	14.2465	3.7745
	Left	2017-02-20	0.866	0.819	0.8425	0.9179
		2021-01-04	2.047	2.012	2.0295	1.4246
		2023-05-26	2.577	2.408	2.4925	1.5788
Patient 4	Right	2016-12-06	0.628	0.575	0.6015	0.7756
		2018-11-27	0.969	0.954	0.9615	0.9806
		2022-06-17	1.270	1.077	1.1735	1.0833
	Left	2016-12-06	0.018	0.018	0.0180	0.1342
		2018-11-27	0.019	0.019	0.0190	0.1378
		2022-06-17	0.019	0.041	0.0300	0.1732
Patient 5	Right	2015-09-04	0.016	0.025	0.0205	0.1432
		2016-09-16	0.045	0.057	0.0510	0.2258
	Left	2015-09-04	1.470	1.445	1.4575	1.2073
		2016-09-16	1.953	1.897	1.9250	1.3874
Patient 6	Right	2022-07-22	0.404	0.382	0.3930	0.6269
		2023-01-13	0.419	0.375	0.3970	0.6301
	Left	2022-07-22	1.918	1.982	1.9500	1.3964
		2023-01-13	2.203	2.128	2.1655	1.4716
Patient 7	Right	2010-12-21	0.020	0.013	0.0165	0.1285
		2020-09-15	0.816	0.728	0.7720	0.8786
	Left	2010-12-21	1.371	1.212	1.2915	1.1364
		2020-09-15	2.267	1.910	2.0885	1.4452
Patient 8	Right	2013-08-16	1.170	1.119	1.1445	1.0698
		2016-11-11	1.842	1.766	1.8040	1.3431
	Left	2013-08-16	0.969	0.842	0.9055	0.9516
		2016-11-11	3.352	3.234	3.2930	1.8147

Patient ID, laterality (right or left eye), and measurement time points are shown. The table includes the atrophic area values (mm^2^) assessed independently by Evaluator 1 and Evaluator 2, as well as the mean values of these measurements (mm^2^). Additionally, the square roots of the mean atrophic areas (mm) are provided to facilitate standardized comparison across eyes and time points.

**Table 3:** AIC values and fixed-effect slope estimates across three mixed-effects models.

Model	AIC Value	Fixed-Effect Slope Estimate (SE)
Model 1: Random intercept for each patient	118.8	0.0636 (0.0526)
Model 2: Random intercept and random slope for each patient	109.2	0.0664 (0.0434)
Model 3: Random intercept and random slope for each eye nested within patient	69.4	0.0677 (0.0126)

The table presents the AIC values and the fixed-effect slope estimates from the three models. SE: standard error.

**Table 4:** Estimated slopes of the square root of the atrophic area from fixed-effects and mixed-effects models.

	Fixed-Effects Model (AIC: 19.2)		Mixed-Effects Model (AIC: 1.7)	
Laterality	Right	Left	Right	Left
Patient 1	0.0195	0.0228	0.0194	0.0227
Patient 2	0.1754	0.263	0.1723	0.2584
Patient 3	0.059	0.108	0.0585	0.1072
Patient 4	0.0525	0.0074	0.052	0.0073
Patient 5	0.0799	0.1741	0.0624	0.136
Patient 6	0.0066	0.1568	0.0029	0.0679
Patient 7	0.0771	0.0317	0.0768	0.0316
Patient 8	0.0844	0.2665	0.082	0.2591

Slopes are expressed as annual change in the square root of the atrophic area and were estimated separately using fixed-effects and mixed-effects models. Data are presented for each eye of each patient. AIC: Akaike Information Criterion.

**Table 5:** Summary of variance components for the square root of the variance ratio in the fixed-effects and mixed-effects models.

	Factor	Degree of Freedom	Sum of Square	Mean Square	Variance Ratio^1/2^
Fixed-effects model (AIC: 19.2)	Eye within patient	8	0.0394	0.0049	0.50
	Patient	7	0.0689	0.0098	
Mixed-effects model (AIC: 1.7)	Eye within patient	8	0.0274	0.0034	0.34
	Patient	7	0.0699	0.0100	

This table compares the relative magnitude of inter-ocular and inter-individual variability. Degrees of freedom, sum of squares, mean square, and the square root of the variance ratio are shown for each factor. ^1/2^: square root transformation of the values. AIC: Akaike Information Criterion.

**Table 6:** Summary of variance components for the square root of the variance ratio in fixed-effects and mixed-effects models, excluding Patient 6.

	Factor	Degree of Freedom	Sum of Squares	Mean Square	Variance Ratio^1/2^
Fixed-effects model (AIC: 22.1)	Eye within patient	7	0.0281	0.0040	0.35
	Patient	6	0.0682	0.0114	
Mixed-effects model (AIC: 6.9)	Eye within patient	7	0.0252	0.0036	0.34
	Patient	6	0.0633	0.0106	

This analysis was conducted to assess the impact of Patient 6 on the variance component and to compare the relative magnitude of inter-ocular and inter-individual variability. Degrees of freedom, sum of squares, mean square and the square root of the variance ratio are shown for each factor. ^1/2^: square root transformation of the values. AIC: Akaike Information Criterion.

## Data Availability

All data analyzed in this study are included in the manuscript. Further inquiries regarding the data may be directed to the corresponding author, T.Y.

## Abbreviations

The following abbreviations are used in this manuscript:

AMD	Age-related macular degeneration
GA	Geographic atrophy
CRA	Chorioretinal atrophy
VEGF	Vascular endothelial growth factor
AIC	Akaike Information Criterion
RPE	Retinal pigment epithelium
CNV	Choroidal neovascularization
